# The relationship between social participation and depressive symptoms among Chinese middle-aged and older adults: A cross-lagged panel analysis

**DOI:** 10.3389/fpubh.2022.996606

**Published:** 2022-10-13

**Authors:** Yifei Ding, Lanshuang Chen, Zhen Zhang

**Affiliations:** ^1^CAS Key Laboratory of Mental Health, Institute of Psychology, Chinese Academy of Sciences, Beijing, China; ^2^Department of Psychology, University of Chinese Academy of Sciences, Beijing, China

**Keywords:** social participation, depressive symptoms, middle-aged and older adults, online participation, offline participation, mental health, mutual influence

## Abstract

**Objectives:**

This study examined the mutual effects of social participation and depressive symptoms in middle-aged and older Chinese adults from offline and online perspectives. Reciprocal relationships between depressive symptoms and the four subtypes of offline participation were also examined.

**Methods:**

Based on China Health and Retirement Longitudinal Study data (CHARLS), this study conducted ID matching for three waves of data from 2013, 2015, and 2018. One thousand nine hundred ninety-nine samples for individuals over 50 were obtained. A series of cross-lagged analyses were conducted to examine the mutual influence of social participation and depressive symptoms. Depressive symptoms were assessed using the short version of the Center for Epidemiologic Studies Depression Scale (CESD-10). The social participation questionnaire included nine items referring to offline participation (eight items) and online participation (one item). Several demographic variables were adjusted when conducting the cross-lagged analyses.

**Results:**

Social and offline participation had significant two-way associations with depressive symptoms. Depressive symptoms had greater impacts on social and offline participation than in the opposite direction. Online participation has a significant one-way impact on depressive symptoms. The relationship between specific types of offline participation and depressive symptoms varied in existence and direction.

**Discussion:**

Overall, there was significant bidirectional causality between social participation and depressive symptoms. Social participation, both offline and online, played a positive role in alleviating depressive symptoms. Depressive symptoms also affected the level of social participation to some extent among middle-aged and older Chinese adults over time.

## Introduction

China has become one of the fastest aging countries in the world. According to the Seventh National Census, the number of people aged 60 or above reached 264 million, accounting for 18.7% of the total population, an increase of 5.4% compared with the Sixth National Census in 2010 (1). Coping with an aging population and improving the quality of life in old age has become a matter of national concern. Depression continues to be one of the most critical mental health problems influencing the quality of life of middle-aged and older adults (2, 3). A recent nationwide epidemiological survey in China indicated that individuals aged 50 years and older had a higher lifetime prevalence of depression than other age groups (3). Maintaining appropriate social participation is particularly important for the mental health of middle-aged and older adults as an essential indicator of active aging (4). A growing body of research has demonstrated that social participation positively contributes to the mental status of middle-aged and older adults. This includes better cognitive function (5), lower risk of mental disorders (6, 7), and higher subjective well-being (8).

In the long run, social participation and mental health become preconditions and consequences of one another. Good physical and mental health is necessary for older adults to participate in social activities. Depressive symptoms negatively affect the possibility and degree of participation in social activities by reducing physical function (9), weakening cognitive ability (10, 11), and increasing the risk of disease (12). Simultaneously, participating in social activities also enhances the mental health of older adults. The positive role of social participation in alleviating depressive symptoms has been confirmed (13–15); however, relatively few studies have examined the simultaneous mutual influence of the two. Thus, the primary concern of the current study was to show the two-way association between social participation and depressive symptoms.

### Social participation and depressive symptoms: Theories and empirical research

Activity theory is one of the most influential theories on aging. It emphasizes that older adults who are more active in participating in various activities will have a better quality of life and well-being and that physical and psychological functions are preconditions for engaging in social activities or social affairs (16). When individuals enter late adulthood, their physical functions, social roles, and social networks undergo adverse changes. Maintaining active social participation is needed to counteract the negative effects of such changes on their mental health. Social participation has been found to slow the process of cognitive decline (5), which protects older individuals from adverse psychological conditions such as depressive symptoms (17, 18). Role continuity or role replacement through active activities may contribute to one's sense of meaning and identity (19, 20), thereby mitigating negative mood and depressive symptoms caused by the interruption of one's original social role (21).

Social capital theory also helps to explain the reciprocal relationship between social participation and depressive symptoms. Social networks, reciprocal norms, and trust are often considered three of the primary constructs of social capital (22, 23). A large body of literature shows that social participation helps maintain or even expand one's social network size as a structural component of social capital. This offers instrumental and psychosocial support and contributes to mitigating depressive symptoms in older adults (24, 25). Social participation can also enhance the cognitive component of social capital, which often refers to trust and reciprocity (26). Hu (27) demonstrated that social participation could enhance people's trust in others, which helps improve individuals' mental health. The social capital perspective provides theoretical support for the predictive effect of depressive symptoms on social participation. Depression, a well-known factor of social isolation, can significantly reduce a person's sense of pleasure, self-efficacy, desire to act and connect with others, shrink their social network, and ultimately decreases opportunities for older adults to participate in society (28–30). Liu et al. (31) showed that depressive symptoms could lead to interpersonal distrust, which prevents people from engaging in normal social activities. In recent years, social capital theory also was applied to understand the effect of online participation on offline health (32). Social capital accumulated by online interactions helps to gain social support and reinforce norms of reciprocity, and further contributes to mental health. In addition, extant studies have shown that depressive symptoms affect cognitive processes and physical function and have a negative impact on self-evaluation. This can affect interpersonal exchange and reciprocal interaction, further hindering social participation (10, 30).

### Deficiencies of extant empirical research

Although numerous studies have shown a strong link between social participation and mental health, including depression, empirical evidence of the causal relationship and direction must be explored further. Most studies often adopt a cross-sectional design and focus on the one-way effect of social participation on depressive symptoms, which partly weakens the causal explanation (7, 33). There are relatively few investigations on reciprocal relationships between social participation and depressive symptoms (29, 34). Sirven and Debrand (29) confirmed that social participation had a causal beneficial impact on mental health and vice versa. However, the effect of mental health on social participation appeared to be significantly higher than vice versa. In addition, some inconsistent findings have been reported. Some studies have not found significant associations between social participation and depression or related psychological symptoms (35, 36). A prospective study found no evidence that social participation reduces the risk of depression (35). Overall, the data limitations and study designs on this issue have led to inconsistent, unconvincing, and biased results.

Social participation is an umbrella term that often covers multiple dimensions and involves various social activities. Offline participation is still the mainstream social activity for older adults. However, as a new form of social participation or communication, online participation is increasing in late adulthood. According to a recent nationwide report, netizens aged 50 years or older account for 28% of the total number of netizens (37). Online participation, such as chatting and visiting social networking sites, is becoming popular among middle-aged and older adults. They use the Internet to keep in touch with existing social relationships, build new ties with social members, and engage in various activities with others (38). Existing studies have preliminarily indicated that online participation can effectively reduce social isolation, loneliness, and depressive symptoms and improve subjective well-being (24, 33, 39).

Meanwhile, online participation helps maintain the social network scale of older adults whose offline social activities decrease with age and declining physical function. It also increases instrumental and emotional support and ultimately benefits their mental health (24, 39). Additionally, online participation helps older adults adapt to changing environments and stress and can further reduce the risk of depression (33, 38). Nevertheless, few studies have directly examined the association between online social participation, social technology use, and depression among middle-aged and older adults in China (40). Thus, online social participation should be fully considered an emerging and indispensable form of social participation in the context of offline social participation.

### Current focus

Using a nationally representative database, the current study attempted to explore the mutual influence of social participation and depressive symptoms among middle-aged and older adults. This study used longitudinal panel data and cross-lagged analyses. Our study combined online and offline participation with four specific types for analysis to provide a more comprehensive perspective of social participation. Based on the above considerations, this study proposes the following hypotheses:

H1: Social participation that integrates offline and online participation negatively predicts depressive symptoms.
H1a: Offline participation negatively predicts depressive symptoms.
H1b: Online participation negatively predicts depressive symptoms.
H2: Depressive symptoms negatively predict social participation.
H2a: Depressive symptoms negatively predict offline participation.
H2b: Depressive symptoms negatively predict online participation.

## Methods

### Data and sample

**Figure 1:**
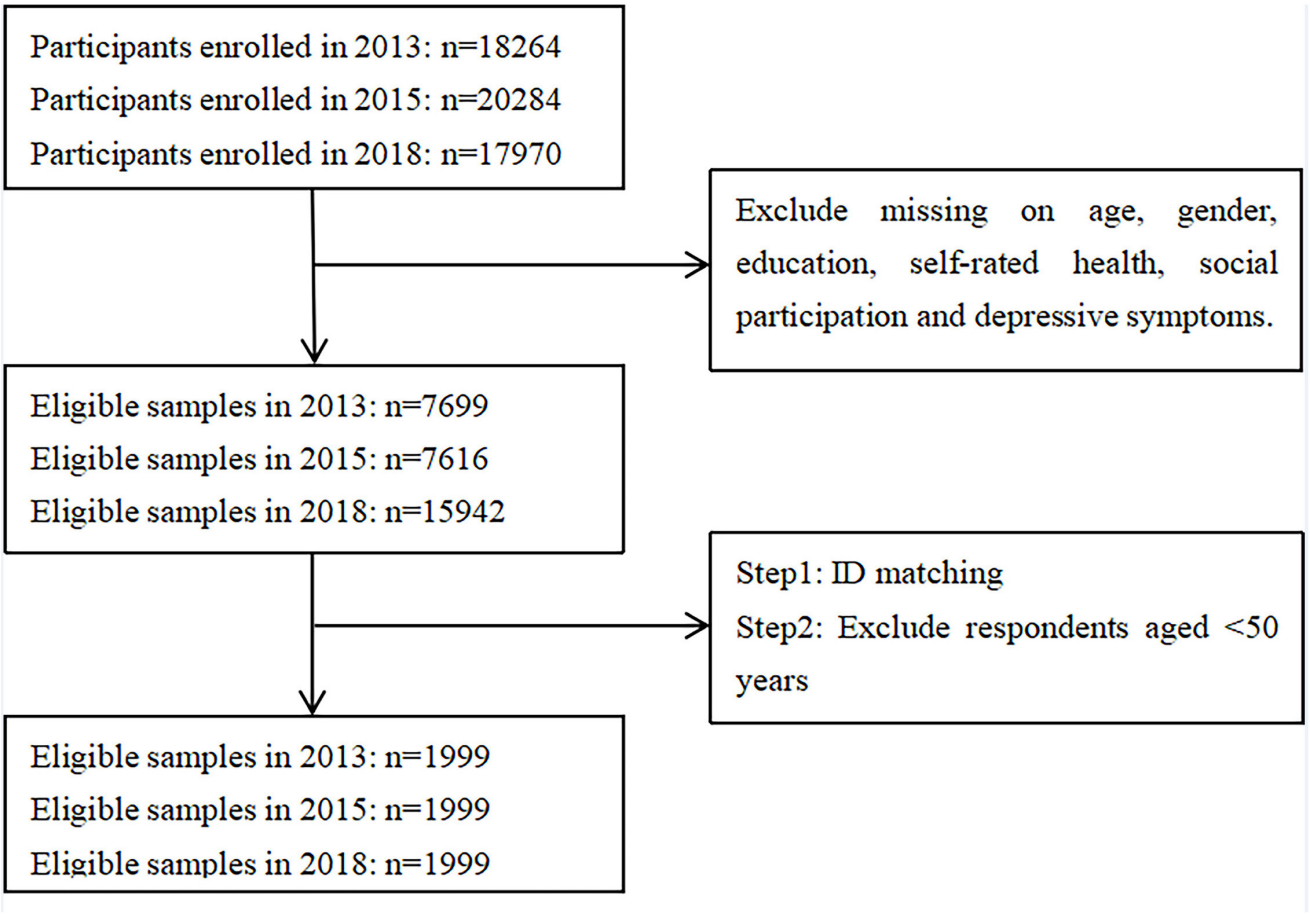
Flowchart of participant selection.

The database used in the current study is the China Health and Retirement Longitudinal Study (CHARLS). This nationally representative longitudinal survey provides high-quality data on Chinese adults aged 45 years and above with a wide range of information, including socioeconomic status and health conditions. China Health and Retirement Longitudinal Study has a large sample size and wide coverage. The survey adopted a multi-stage, stratified, probability proportional to size (PPS) random sampling method and involved residents from 150 counties and 450 villages across 28 provinces in China (41). The baseline survey was conducted in 2011 and covered 17,708 individuals, with a response rate of 80.51%. Three follow-up surveys were conducted on 18,264, 20,284, and 17,970 individuals. Cross-sectional response rates were 82.63%, 82.13%, 83.84% in 2013, 2015, and 2018, respectively (41). The current study uses data from CHARLS 2013, 2015, and 2018 (41). The 2013 data served as the baseline, primarily due to the low number of online participants in China in 2011. We selected participants aged 50 years and above with no missing answers to the questions on social participation, depressive symptoms, age, gender, education, and self-rated health in each wave. We ultimately obtained 1999 participants through ID matching (Figure 1). Taking the data of 2018 as an example, we conducted relevant χ^2^-tests and found that the differences in demographic variables as covariates between selected samples and unselected samples were insignificant or relatively small (gender: χ^2^ = 0.195, *p* = 0.659; self-rated health: χ^2^ = 7.751, *p* = 0.101; education: χ^2^ = 10.522, *p* = 0.032). Given similar demographic structure of the two samples, we infer that there was no serious sample bias.

### Measurements

#### Social participation

There was one question in CHARLS asking about SP entitled, “Have you done any of these social activities in the past month?” If the participants participated in at least one of the following social activities, then they were considered to “have social participation (SP)”: (1) interacted with neighbors or friends; (2) played ma-jong, chess, cards, or went to a community club; (3) provided help to family, friends, or neighbors who did not live with you and did not pay for the help; (4) went to a sport, social, or another kind of club; (5) took part in a community-related organization, (6) did voluntary or charity work; (7) cared for a sick or disabled adult who does not live with you; (8) attended an educational or training course; (9) used the Internet. If they participated none of the above activities, they were considered to “not have social participation.” If the participants answered “yes” to question (9), they were considered to “have online participation (ONP).” If the participants joined in at least one of the activities in questions (1)–(8), they were considered to “have offline participation (OFP).” According to the existing literature (14, 15), OFP was divided into four specific types in this study. Questions (4) and (5) were named club activities (CLA), and questions (2) and (8) were named intellectual activities (INA). Question (1) was classified as simple interpersonal activities (SIA), and questions (3), (6), and (7) were classified as volunteer activities (VOA).

#### Depressive symptoms

The CESD-10, a short form of the Center for Epidemiologic Studies Depression Scale, was used to measure depressive symptoms in CHARLS participants (42). The scale comprises 10 items, with answers encoded on a four-point scale ranging from 0 to 3. All items were added, with a total score ranging from 0 to 30. A higher score indicates higher levels of depressive symptoms. The scale has been widely used in middle-aged and older Chinese adults and has good internal validity (43).

### Covariates

In the current study, sociodemographic factors include age (in years), gender (1 = male, 2 = female), education (1 = illiterate or without primary school education, 2 = elementary school, 3 = middle school, 4 = high school, 5 = college or university and above), and self-rated health (1 = very poor, 2 = poor, 3 = fair, 4 = good, 5 = very good). Previous studies have shown that gender, education, and self-rated health are three important factors influencing mental health in late adulthood (8, 44). Therefore, we controlled for the roles of gender, education, and self-rated health in 2013 data in the following cross-lagged analyses.

### Statistical analyses

**Figure 2:**
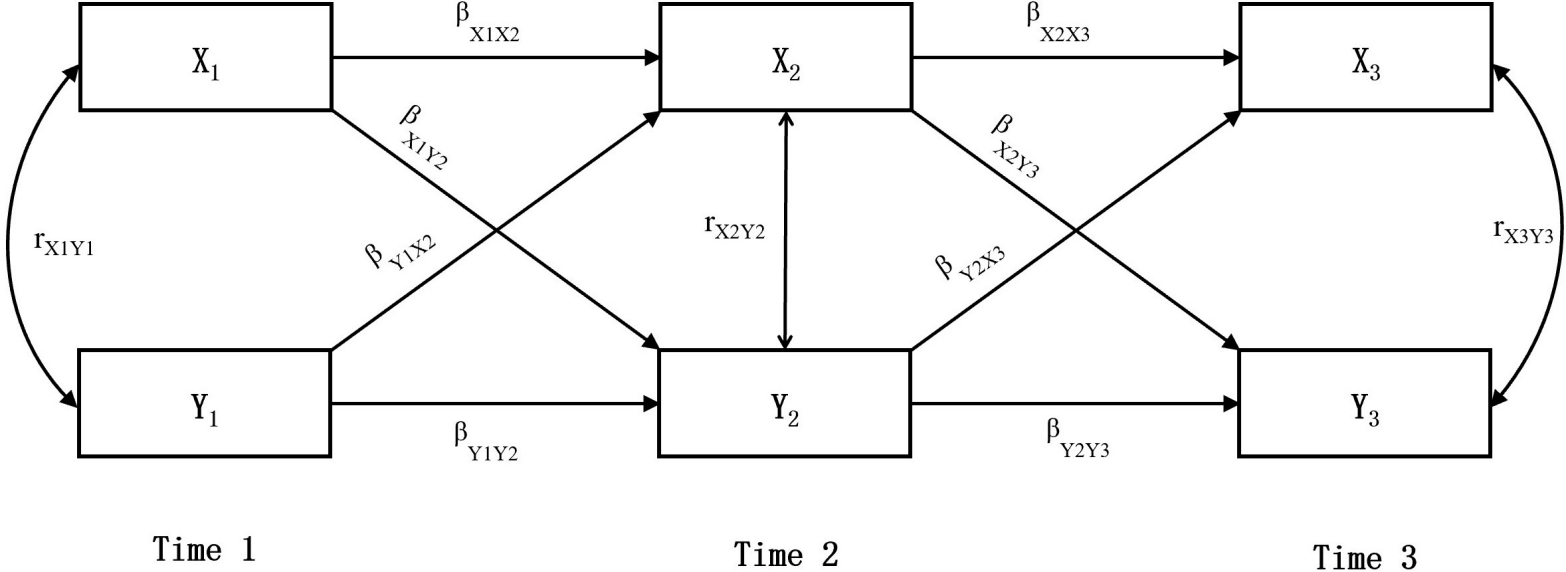
Cross-lagged panel analysis.

SPSS26.0 was used for descriptive statistics and correlation analyses. Mplus8.3 and Maximum Likelihood Estimation (MLE) were used to conduct the cross-lagged and model fit analyses. Several indices evaluated the goodness of fit, including χ^2^/df, the comparative fit index (CFI), standardized root mean square residual (SRMR), and root mean square error of approximation (RMSEA) (45). Cross-lagged panel analysis is mainly used to describe the mutual influences between variables over time, which is considered as an effective analysis method to probe potential causality between variables. The most basic cross-lagged panel model includes two constructs measured at two time points, namely, is to compare the relationship between variables X at Time 1 and variable Y at Time 2 with the relationship between variable Y at Time 1 and X at Time 2 when controlling for correlations between variables within time-points and autoregressive effects across time. This basic model can be extended to three or more rounds of research (46). Taking the three-round cross-lagged panel model in this study as an example (Figure 2), if β_X1Y2_ and β_X2Y3_ are significant, it means that X affects Y; If β_Y1X2_ and β_Y2X3_ are significant, it means that Y affects X; If β_X1Y2_, β_X2Y3_, β_Y1X2_, and β_Y2X3_ are all significant, it means that X and Y have reciprocal influences on each other, and the direction with a larger β coefficient has a stronger influence. In this study, basic demographic variables at baseline (T1) were controlled in the cross-lagged model, and three rounds of data were used for cross-lagged analysis.

## Results

### Descriptive analyses

A total of 1,999 samples were obtained after three waves of data matching: 45.7% were aged 50–59 and 54.3% were over 60 years old (M = 61.09, SD = 7.54); 49.6% were males and 50.4% females; 39.6% illiterate or without primary school education, 22.9% elementary school, 37.5% middle school and above; 24.7% self-rated poor and very poor health and 75.3% fair and better (Table 1).

**Table 1:** Demographic characteristics of the participants at baseline (*N* = 1,999).

**Variable**		***n* (%)**
Age (year)	50–54	453 (22.7%)
	55–59	456 (23.0%)
	60–64	467 (23.5%)
	65–69	325 (16.4%)
	70–91	298 (14.4%)
Gender	Male	992 (49.6%)
	Female	1,007 (50.4%)
Education	Illiterate or without primary school education	792 (39.6%)
	Elementary school	458 (22.9%)
	Middle school	455 (22.8%)
	High school	243 (12.2%)
	College or university and above	51 (2.5%)
Self-rated health	Very poor	95 (4.8%)
	Poor	397 (19.9%)
	Fair	1,069 (53.5%)
	Good	265 (13.3%)
	Very good	173 (8.5%)

The statistical results of the three waves of SP showed that the number of people who participated in offline activities was generally slightly more than those who did not. Those participating in online activities were the opposite. Offline participation tended to decline with age, and the four specific types of offline participation showed similar trends. In contrast, the survey's online participation rate increased steadily (Supplementary Tables S1, S2).

According to Andersen et al. (42), a CESD-10 score of 10 was used as the cutoff point to identify depressive symptoms in older adults. The findings in the three waves outlined a high rate of depressive symptoms in middle-aged and older Chinese people. The average prevalence was approximately 40% and showed an upward trend over time (Supplementary Table S3). The prevalence rate increased from 36.4% to 43.3%.

### Correlation analyses

Correlation analyses showed significant cross-sectional and longitudinal correlations between SP, including offline and online participation, and depressive symptoms (Supplementary Table S4).

### Model fit statistics

According to the standards of model and data fitting, CFI > 0.9, RMSEA < 0.08, and SRMR < 0.08 are the criteria for the goodness of fit (45). The three models of SP, online and offline, all had a good fit. The fit results of the four specific offline activity models were all satisfactory (Supplementary Tables S5, S6).

### Cross-lagged panel analyses

**Figure 3:**
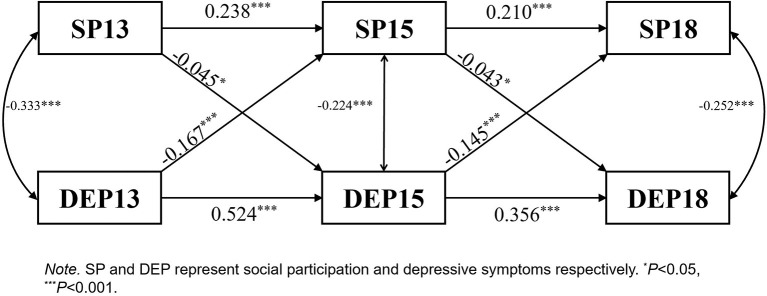
SP↔DEP cross-lagged panel.

*SP*↔*DEP:* SP and DEP showed a significant two-way negative relationship. DEP had a greater effect on SP than in the opposite direction (Figure 3).

**Figure 4:**
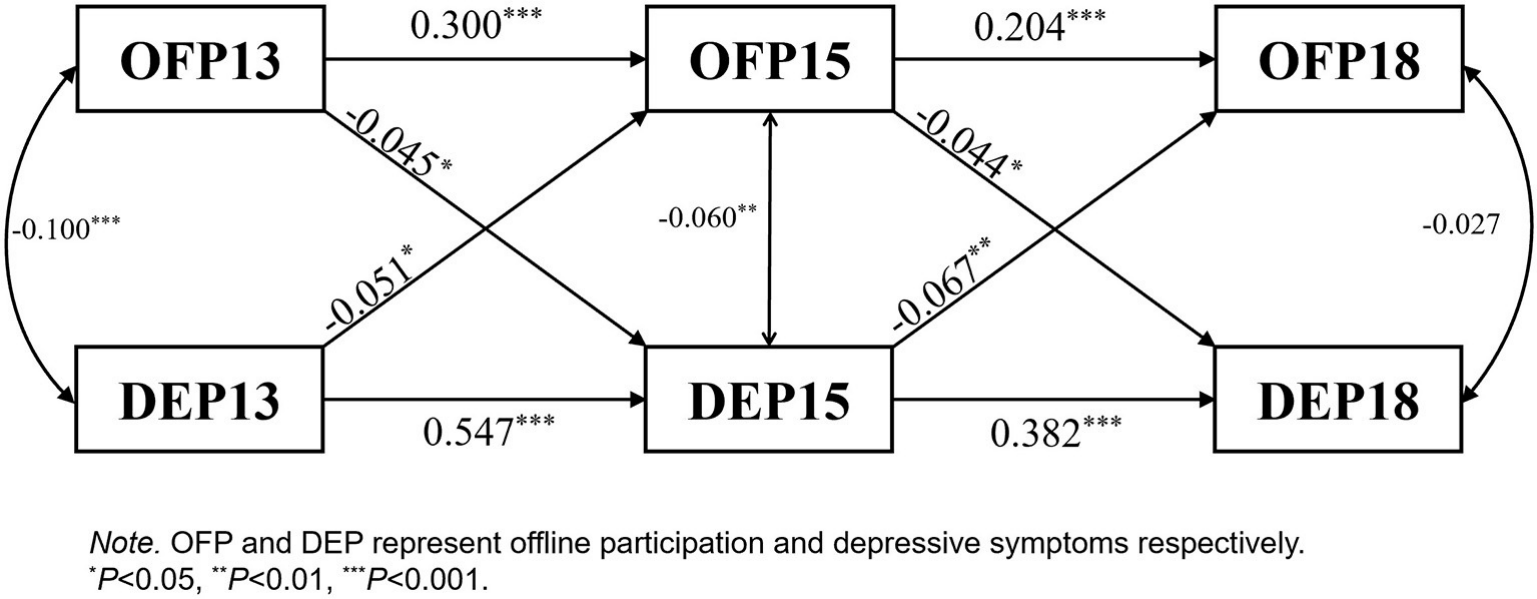
OFP↔DEP cross-lagged panel.

*OFP*↔*DEP:* OFP and DEP showed a significant two-way negative relationship. DEP had a greater effect on OFP than in the opposite direction (Figure 4).

**Figure 5:**
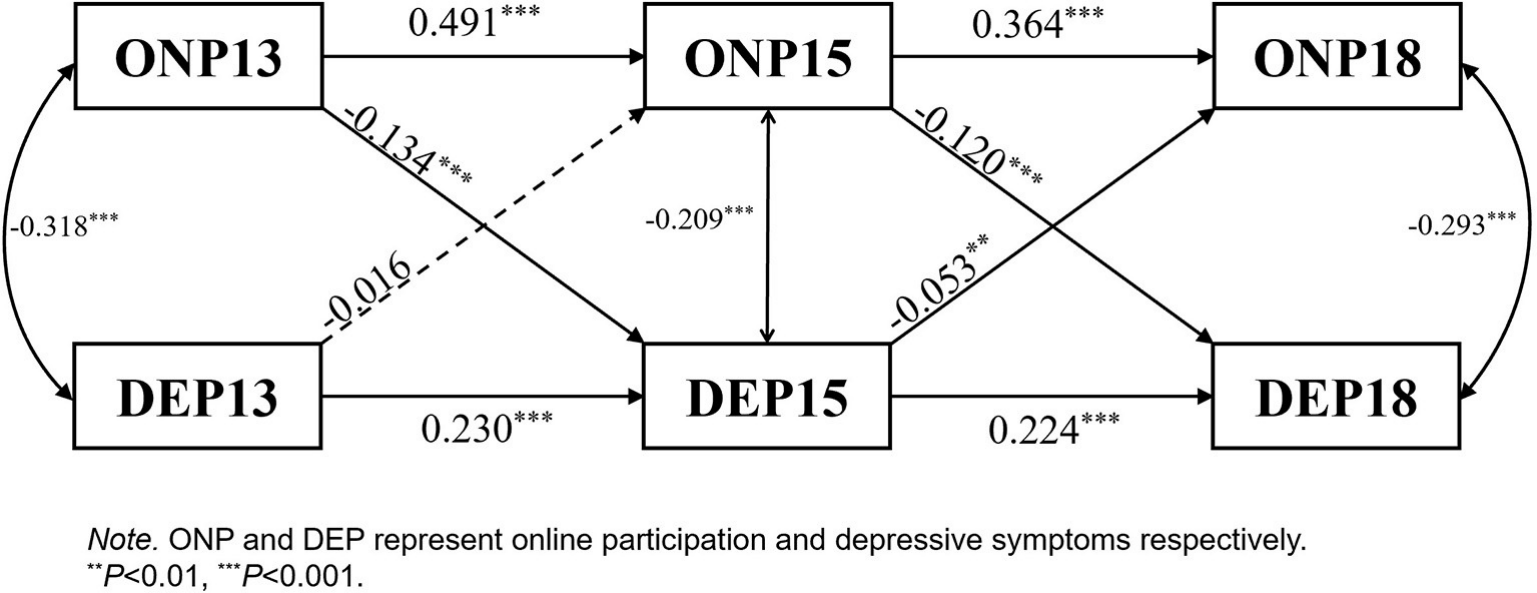
ONP↔DEP cross-lagged panel.

*ONP*↔*DEP:* The lagged effect of ONP on DEP is significant. DEP13 had no significant negative effect on ONP15. However, the negative effect of DEP15 on ONP18 was significant (Figure 5).

*Specific OFP*↔*DEP:* CLA and DEP showed a significant two-way negative relationship. The lagged effect of DEP on INA was significant, whereas the lagged effect of INA on DEP was negative but not significant. No significant lagged effects were found between SIA and DEP and between VOA and DEP (Supplementary Figures S1–S4).

## Discussion

Using a nationally representative database across three waves of surveys, this study examined the reciprocal relationship between SP and depressive symptoms among middle-aged and older adults in China. The current study also explored the associations between depressive symptoms and two primary forms of SP: offline and online. Furthermore, the reciprocal relationships between four specific types of offline activities and depressive symptoms were analyzed.

### Social participation

The three survey waves showed that nearly half of middle-aged and older adults surveyed did not participate in any social activities, and the participation rate declined slightly with successive waves. This finding reflects the reality that the social functioning of older adults decreases with age (47). A recent study indicated that the proportion of older adults participating in social activities in China was relatively low (14). Offline participation remains the most important and easily accessible form of SP among middle-aged and older adults. More specifically, SIA were the most common form of offline participation, while club and volunteer activities had lower participation rates. The reason may be that participating in volunteer or club activities in a group requires more active motivation, social resources, and better organizational management.

Online participation—an emerging form of social participation—showed a rising trend, although the proportion of online participants was still relatively small. With the popularity of the Internet in China, middle-aged and older adults have more opportunities to access it. They can browse the news, search for information, chat, make friends, and watch short social videos. Online participation has become a beneficial supplement to offline participation (39). Online participation as a platform for interpersonal interactions can overcome the barriers caused by distance and time, enabling people to maintain their social networks, even when they are physically disengaged from offline activities (48, 49).

### Depressive symptoms

According to a recent epidemiological survey, the lifetime prevalence rate of depressive disorders in China had reached 6.9%. People aged 50 years and older had a significantly higher prevalence rate, accounting for 53% of the total patients (3). The proportion of depressive symptoms was more than 36% in each wave and showed an increasing trend, which was similar to previous findings (15). The current study confirmed that middle-aged and older adults in China are at a relatively higher risk of depressive symptoms.

### Relationship between SP and depressive symptoms

There was a bidirectional relationship between SP and depressive symptoms; H1 and H2 were therefore confirmed. Meanwhile, depressive symptoms had a greater impact on SP than in the opposite direction, consistent with a previous study (29). Social participation can alleviate depressive symptoms; more remarkably, depressive symptoms can inhibit future SP.

Similar to general SP, offline participation had a significant two-way relationship with depressive symptoms; therefore, H1a and H2a were confirmed. Depressive symptoms have a greater effect on offline participation than in the opposite direction. The four types of offline activities varied in their association with depressive symptoms. Club activity and depressive symptoms showed a significant bidirectional association. Depressive symptoms demonstrated a significant negative effect on intellectual activities, but there were no significant associations with simple interpersonal and volunteer activities. Club activities included organized activities for physical exercise or entertainment. Numerous studies have shown that physical exercise can improve health and cognitive ability, thereby reducing the risk of depression in older adults (14, 50, 51). Additionally, social membership provided by clubs enables older adults to obtain more social resources, social support, and a sense of belongingness. These factors play a protective role against depressive symptoms (19, 20). Depressive symptoms have negative effects on physical health and social function, significantly affecting one's ability and willingness to participate in social activities in an organization (28, 30). Intellectual activities mainly included playing mahjong or chess and participating in educational or training courses. Previous studies have shown that depressive symptoms can lead to cognitive impairment, affecting people's ability to engage in brain-related activities (10, 11). Simple interpersonal activities primarily involve interactions with neighbors or friends. This study did not detect a significant relationship between SIA and depressive symptoms. This may be due to the dramatic change in people's lifestyles with the acceleration of urbanization in China (52), where door-to-door interactions with neighbors or friends have become less common compared to previous decades. The current study indicated that SIA decreased significantly with the waves. In addition, previous studies have shown that more advanced skills and related activities have a more significant impact on the mental health of older adults (14, 53). Simple interpersonal activities do not require high physical strength, intelligence, and social connections; therefore, their effect on depressive symptoms is limited. Volunteer activities included helping and taking care of people who were separated from them, as well as charitable activities. This study did not find a significant effect of volunteer activities on depressive symptoms, consistent with recent studies (14, 15, 51). In China, the number of people involved in volunteer activities is relatively low. Most older adults need to care for their grandchildren, which consumes considerable time and energy. In addition, the emotional benefit of volunteer activities may be offset mainly by the sense of obligation and feelings of fatigue involved in caring for or helping others (54). Above all, inferences must be further tested through future empirical studies.

Online participation presented a one-way relationship with depressive symptoms, consistent with a recent study (33). In the current study, online participation played a role in buffering and preventing depressive symptoms; therefore, H1b was confirmed. People can keep in touch with their families and relatives and build new weak ties with acquaintances or casual strangers through the Internet, regardless of physical conditions and spatiotemporal limitations. Online participation also provides people in late adulthood with a new way to relax and enrich their lives (24). The social support and spiritual satisfaction obtained from the Internet can help them effectively alleviate negative psychological experiences, such as depressive symptoms. H2b was not confirmed in this study; however, there was still a trend for depressive symptoms to negatively predict online participation. The negative effect of depressive symptoms in the 2015 wave on online participation in 2018 was significant. The reason for not testing for the cross-lagged negative effect between depressive symptoms and online participation may be that the Internet was not popularized in China in 2013, and the number of online participants was relatively small. Furthermore, the purpose of online participation may have changed. In 2013, fewer older adults had access to the Internet, most of them using it for work purposes. With the popularity of the Internet, the aim of online participation has shifted to interpersonal communication.

In general, interactions during SP can sustain social networks and stimulate mutual support under the umbrella of the social capital theory, which will have a positive impact on alleviating depressive symptoms (24, 25, 55). In addition, the sense of belongingness, social identity, trust, personal mastery, or accomplishment obtained from SP can help mitigate depressive symptoms and other adverse psychological experiences in middle-aged and older people (19, 20, 27). Moreover, the shrinking social network, reduced trust, and lack of positive emotions caused by depressive symptoms seriously impede the SP of middle-aged and older adults (29–31).

### Significance and limitations

This study confirmed bidirectional causality between SP and depressive symptoms, suggesting that there may be a virtuous cycle process between SP and mental health in the long run—one is a prerequisite for the other. The positive role of SP in the active aging process should be fully considered without underestimating the negative impact of depressive symptoms on social function. The current findings enrich relevant social capital theory and activity theory. Based on the above findings, SP contributes to the accumulation and increase in social capital, such as social networks and social trust, and improves mental health. Third, the methodological advantages of the study are noteworthy. Immense and nationally representative samples, longitudinal data, and cross-lagged analyses make it possible to explore two-way predictive relationships and obtain robust results. Furthermore, this study conducts multidimensional analyses by combining offline participation with online participation, expanding the scope of SP. Online social communication is also beneficial for maintaining mental health, especially in declining physical conditions, frequent population movements, and a global pandemic that has yet to be fully controlled. With the popularization of Internet use, the role of online participation and communication will receive more attention in future studies. Finally, the practical and policy implications of these findings are discussed. Participation is considered one of the three pillars of active aging. From a micro perspective, SP, including online and offline, should be encouraged, given its protective effect on depressive symptoms. From a macro policy perspective, the current study provides a basis for developing relevant policies and measures to actively cope with aging. Local governments and social organizations should be aware of the importance of social participation in promoting the mental health of retired residents. Based on above findings, it is necessary to adopt measures to promote different forms of social participation, which include creating conditions for communities to organize various forms of offline social activities, and developing social network sites or online communities suitable for middle-aged and elderly groups. Especially during the COVID-19, online social participation has played a positive role in alleviating the shrinkage of social network and loneliness caused by reduced offline interaction. Middle-aged and older adults should be appropriately encouraged to learn and use the Internet, while more social network environments and information content suitable for this group should be created so that they can actively adapt to the development of modern society and improve their physical and mental health. At the same time, this study also provides an inspiration for the clinical intervention of depressive symptoms. For example, clinical practitioners and caregivers should create environment conducive to the middle-aged and elderly people with depressive symptoms to participate in various social activities, activate their participation motivation, and reduce their sense of helplessness. With the popularization and development of Internet technology, patients with depressive symptoms can receive professional treatment and help by joining professional Wechat mutual help groups in a free, safe, and harmonious online space, so as to achieve a dynamic and continuous intervention effects.

This study had some limitations. Firstly, although we used data from the CHARLS database, the number of online participants in the early stage was still low, which may have affected the results of this study. Future studies should continue to investigate the bidirectional causality between depression and online participation. Secondly, there was only one item in this study measuring online social participation, which fails to distinguish its specific content. Follow-up research should be conducted based on further improvement of the database questionnaire in the future. Thirdly, as an authoritative national database in China, CHARLS' age distribution is representative to some extent. Compared to the age distribution of middle aged and older adults in the general population of China, the majority of respondents to CHARLS were slightly younger, possibly because a quite proportion of the elderly were unable to participate in the survey for various reasons, including suffering from more chronic disease, declining physical functions and worse cognitive abilities. Fourthly, this study only considered a small number of demographic variables as covariates, but there are other variables that may affect or confound this relationship between main variables. Life environment, such as family, neighborhood, and community environmental factors, may influence social participation, depression, and the relationship between social participation and depression (56). Thus, more covariates should be included in future studies for examining more accurate and purer mutual relationship of social participation and depressive symptoms. In addition, this study does not discuss the internal mechanism of bidirectional relations between SP and depressive symptoms, which encourages an in-depth exploration of this issue in future studies.

## Conclusion

This study confirms a significant bidirectional relationship between SP and depressive symptoms. Social participation, both offline and online, played a positive role in alleviating depressive symptoms. Depressive symptoms could also affect subsequent SP among Chinese middle-aged and older adults.

## Data availability statement

The original contributions presented in the study are included in the article/Supplementary material, further inquiries can be directed to the corresponding author.

## Ethics statement

The studies involving human participants were reviewed and approved by Scientific Research Ethics Committee, Institute of Psychology, Chinese Academy of Sciences. The patients/participants provided their written informed consent to participate in this study.

## Author contributions

YD collected the data, performed main statistical analyses, and wrote the article. LC contributed to revising the article and statistical analyses. ZZ planned the study, supervised the data analysis, and wrote the article. All authors contributed to the article and approved the submitted version.

## Funding

This work was supported by the grants of the National Natural Science Foundation of China (grant number 71774157), CAS Engineering Laboratory for Psychological Service (grant number KFJ-PTXM-29), and Open Research Fund of the CAS Key Laboratory of Behavioral Science, Institute of Psychology (grant number Y5CX052003).

## Conflict of interest

The authors declare that the research was conducted in the absence of any commercial or financial relationships that could be construed as a potential conflict of interest.

## Publisher's note

All claims expressed in this article are solely those of the authors and do not necessarily represent those of their affiliated organizations, or those of the publisher, the editors and the reviewers. Any product that may be evaluated in this article, or claim that may be made by its manufacturer, is not guaranteed or endorsed by the publisher.
