# Supplementary material for: The relationship between social participation and depressive symptoms among Chinese middle-aged and older adults: A cross-lagged panel analysis
Source: Front Public Health. 2022 Oct 13;10:996606. doi: 10.3389/fpubh.2022.996606 (PMC9608247; doi:10.3389/fpubh.2022.996606)
Supplement: Supplementary file 1 [file Data_Sheet_1.docx]

Table S1 Participation rates of four offline activities（%）

| Variable | 2013 | 2015 | 2018 |
| --- | --- | --- | --- |
| CLA  INA  SIA  VOA | 12.2  25.1  44.5  16.6 | 10.3  22.5  36.4  17.8 | 8.2  19.2  33.0  14.1 |

*Note.* CLA, INA, SIA and VOA represent club activities, intellectual activities, simple interpersonal activities and volunteer activities respectively.

Table S2 Participation rate（*N*=1999）

| Variable | | *n* (%) |
| --- | --- | --- |
| SP13  ONP13  OFP13  SP15  ONP15  OFP15  SP18  ONP18  OFP18 | Yes  No  Yes  No  Yes  No  Yes  No  Yes  No  Yes  No  Yes  No  Yes  No  Yes  No | 1221(61.1%)  778(38.9%)  86(4.3%)  1913(95.7%)  1208(60.4%)  791(39.6%)  1119(56.0%)  880(44.0%)  105(5.3%)  1894(94.7%)  1087(54.4%)  912(45.6%)  1056(52.8%)  943(47.2%)  188(9.4%)  1811(90.6%)  977(48.9%)  1022(51.1%) |

*Note.* SP, ONP, OFP represent social participation, online participation and offline participation respectively.

Table S3 Rate of depressive symptoms（*N=*1999）

| Variable | | *n* (%) |
| --- | --- | --- |
| DEP13  DEP15  DEP18 | YES  NO  YES  NO  YES  NO | 728(36.4%)  1271(63.6%)  792(39.6%)  1207(60.4%)  865(43.3%)  1134(56.7%) |

*Note.* DEP represents depressive symptoms.

Table S4 Inter-correlations among variables

| Variables | 1 | 2 | 3 | 4 | 5 | 6 | 7 | 8 | 9 | 10 | 11 |
| --- | --- | --- | --- | --- | --- | --- | --- | --- | --- | --- | --- |
| 1 SP13 | 1 |  |  |  |  |  |  |  |  |  |  |
| 2 ONP13 | 0.169^***^ | 1 |  |  |  |  |  |  |  |  |  |
| 3 OFP13 | 0.986^***^ | 0.141^***^ | 1 |  |  |  |  |  |  |  |  |
| 4 DEP13 | -0.114^***^ | -0.098^***^ | -0.117^***^ | 1 |  |  |  |  |  |  |  |
| 5 SP15 | 0.317^***^ | 0.153^***^ | 0.313^***^ | -0.095^***^ | 1 |  |  |  |  |  |  |
| 6 ONP15 | 0.133^***^ | 0.602^***^ | 0.117^***^ | -0.089^***^ | 0.209^***^ | 1 |  |  |  |  |  |
| 7 OFP15 | 0.307^***^ | 0.085^***^ | 0.308^***^ | -0.087^***^ | 0.968^***^ | 0.108^***^ | 1 |  |  |  |  |
| 8 DEP15 | -0.111^***^ | -0.086^***^ | -0.115^***^ | 0.569^***^ | -0.119^***^ | -0.090^***^ | -0.112^***^ | 1 |  |  |  |
| 9 SP18 | 0.277^***^ | 0.166^***^ | 0.278^***^ | -0.093^***^ | 0.282^***^ | 0.160^***^ | 0.267^***^ | -0.108^***^ | 1 |  |  |
| 10 ONP18 | 0.113^***^ | 0.481^***^ | 0.103^***^ | -0.114^***^ | 0.158^***^ | 0.470^***^ | 0.116^***^ | -0.136^***^ | 0.304^***^ | 1 |  |
| 11 OFP18 | 0.278^***^ | 0.108^***^ | 0.280^***^ | -0.081^***^ | 0.278^***^ | 0.093^***^ | 0.277^***^ | -0.099^***^ | 0.924^***^ | 0.120^***^ | 1 |
| 12 DEP18 | -0.096^***^ | -0.112^***^ | -0.094^***^ | 0.484^***^ | -0.114^***^ | -0.112^***^ | -0.102^***^ | 0.538^***^ | -0.103^***^ | -0.141^***^ | -0.094^***^ |

*Note.* ^***^*P*<0.001. SP, ONP, OFP and DEP represent social participation, online participation, offline participation and depressive symptoms respectively.

Table S5 Model fit statistics of social participation (online and offline)

| Model | χ^2^ | *df* | χ^2^/*df* | CFI | SRMR | RMSEA |
| --- | --- | --- | --- | --- | --- | --- |
| SP*↔*DEP  ONP*↔*DEP | 46.846  57.290 | 10  10 | 4.685^***^  5.729^***^ | 0.983  0.980 | 0.029  0.024 | 0.073  0.065 |
| OFP*↔*DEP | 69.329 | 10 | 6.933^***^ | 0.971 | 0.030 | 0.062 |

*Note.* SP, ONP, OFP and DEP represent social participation, online participation, offline participation and depressive symptoms respectively.

Table S6 Model fit statistics of four offline activities

| Model | χ^2^ | *df* | χ^2^/*df* | CFI | SRMR | RMSEA |
| --- | --- | --- | --- | --- | --- | --- |
| CLA*↔*DEP | 24.569 | 10 | 2.457^***^ | 0.990 | 0.014 | 0.075 |
| INA*↔*DEP  SIA*↔*DEP  VOA*↔*DEP | 68.954  75.130  72.562 | 10  10  10 | 6.895^***^  7.513^***^  7.256^***^ | 0.980  0.966  0.964 | 0.032  0.031  0.031 | 0.062  0.065  0.064 |

*Note.* CLA, INA, SIA and VOA represent club activities, intellectual activities, simple interpersonal activities and volunteer activities respectively.
