# Supplementary figures and images for: The relationship between social participation and depressive symptoms among Chinese middle-aged and older adults: A cross-lagged panel analysis
Source: Front Public Health. 2022 Oct 13;10:996606. doi: 10.3389/fpubh.2022.996606 (PMC9608247; doi:10.3389/fpubh.2022.996606)

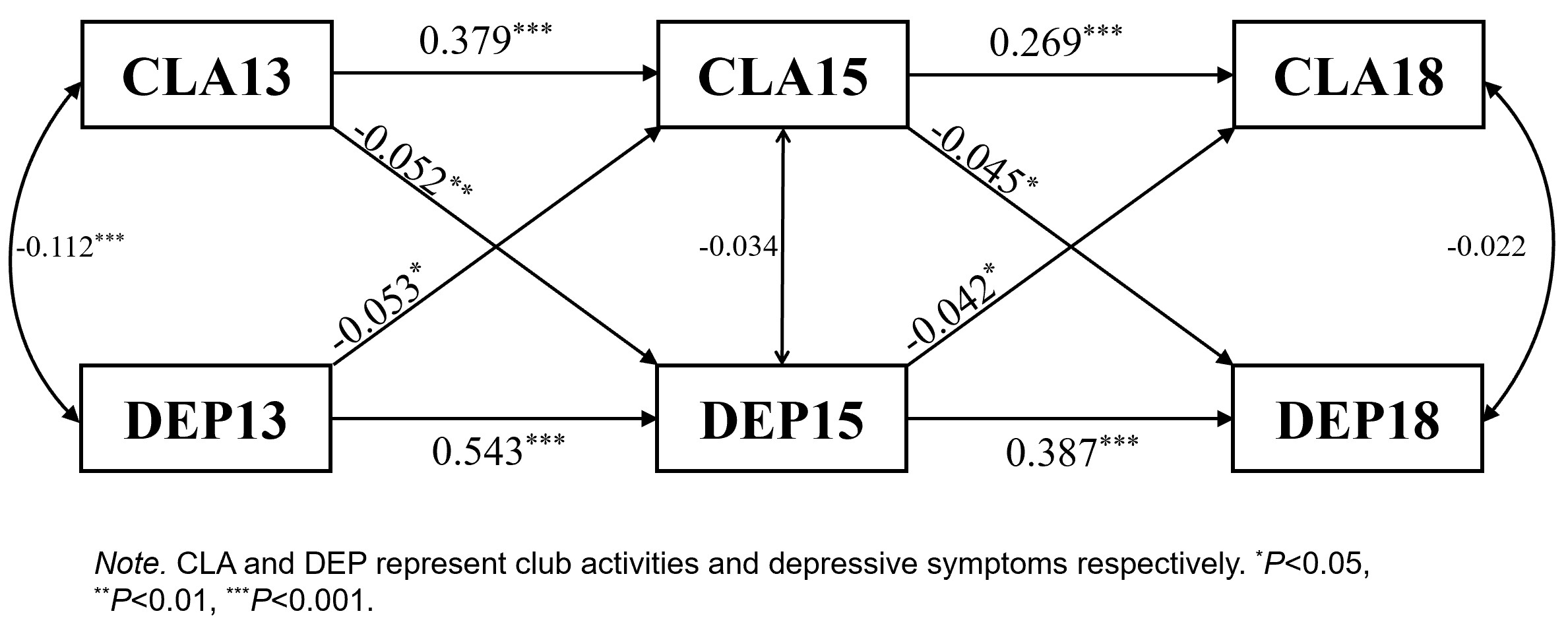

Supplement: Supplementary file 2 [file Image_1.jpg]

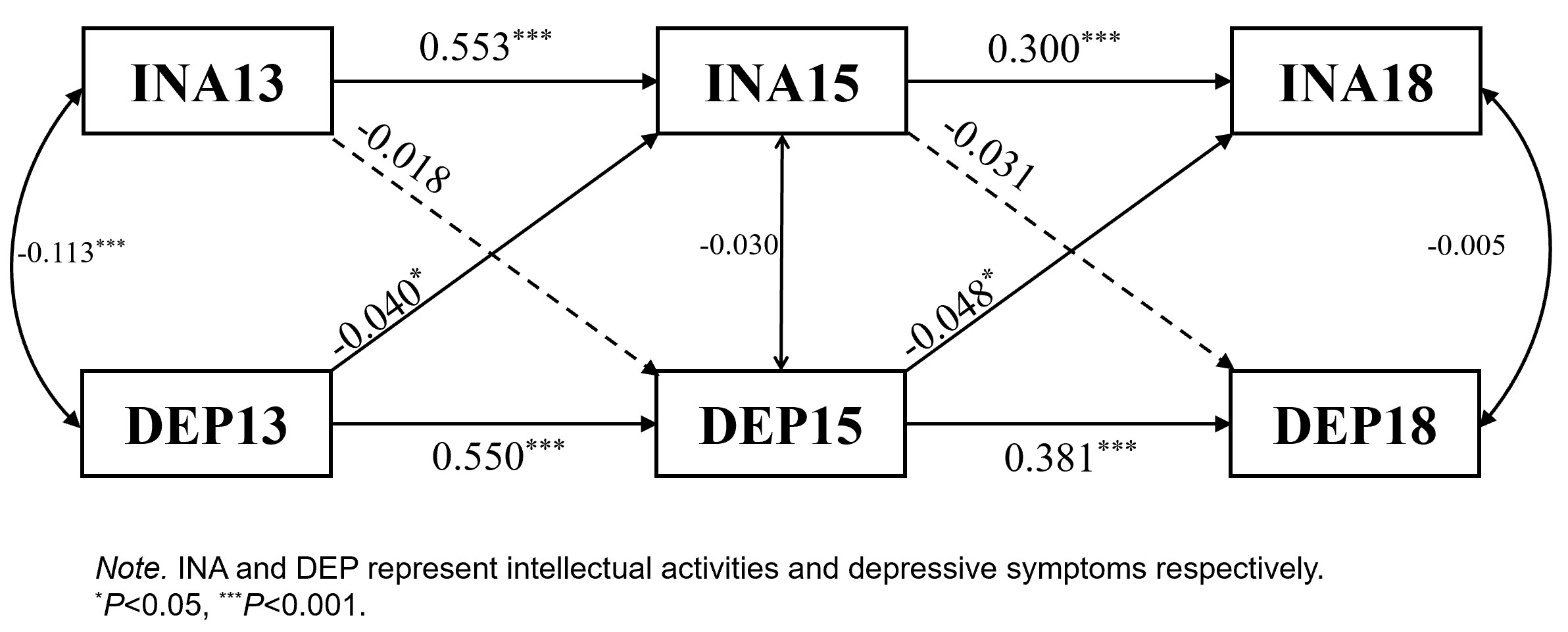

Supplement: Supplementary file 3 [file Image_2.jpg]

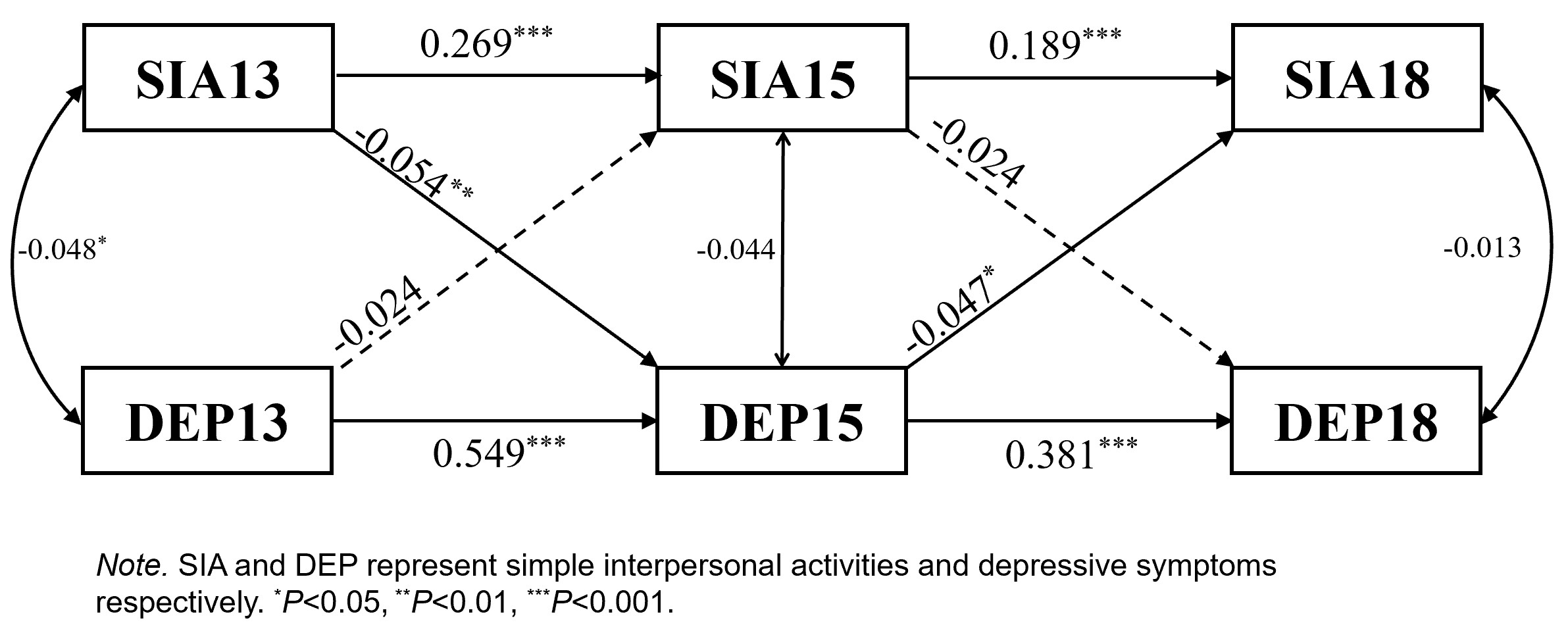

Supplement: Supplementary file 4 [file Image_3.jpg]

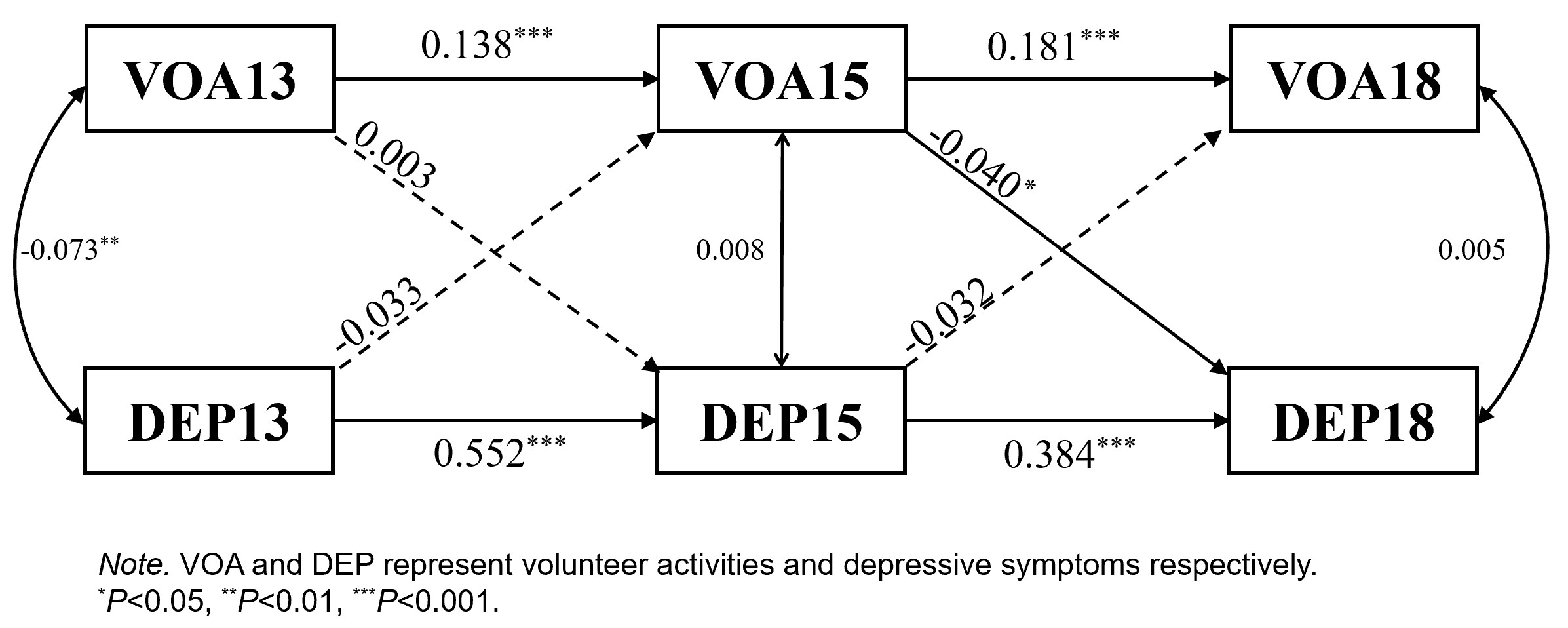

Supplement: Supplementary file 5 [file Image_4.jpg]
